# Figure-8 Tachycardia Confined to the Anterior Wall of the Left Atrium

**Published:** 2004-07-01

**Authors:** Ioan Liuba, Anders Jönsson, Hakan Walfridsson

**Affiliations:** Department of Cardiology, Heartcenter, University Hospital Linköping, Linköping, Sweden

**Keywords:** atrial tachycardia, mapping, catheter ablation

## Abstract

Incisional atrial tachycardias have been described most frequently in patients with previous corrective surgery for congenital heart defects and mitral valve disease. Less information is available on atrial tachycardias appearing late after isolated aortic valve surgery. We report the case of a patient who developed a left figure-8 tachycardia after undergoing aortic valve replacement. During electrophysiologic study the entire cycle length of the tachycardia was mapped within a low voltage area confined to the left anterior atrial wall. However, during ablation a transmural lesion could not be attained. The mapping and ablation strategy along with the mechanism of the tachycardia are discussed.

## Case report

A 57-year-old man with dilated cardiomyopathy and chronic heart failure, who had undergone aortic valve replacement 26 years ago, was referred to our department for a third attempt at radiofrequency (RF) catheter ablation of an atrial tachycardia. According to reports, during the previous two electrophysiologic (EP) studies both a typical atrial flutter and a left atrial reentrant tachycardia (cycle length 190 ms) could be induced with burst pacing. Nevertheless, RF catheter ablation had failed in both cases, either because it was not possible to attain bidirectional block across the cavotricuspid isthmus (despite a large number of RF applications), or due to transformation of left reentrant tachycardia into atrial fibrillation during entrainment mapping and the impossibility of reinducing the same tachycardia after DC cardioversion. The patient continued to have frequent, prolonged episodes of palpitations despite several antiarrhythmic drug trials. Twelve-lead ECG documented an atrial tachycardia with a p-p interval of 240-280 ms and a variable degree of AV block as well as atrial fibrillation. The patient underwent several successful DC cardioversions for both arrhythmias. Transthoracic echocardiography revealed left atrial and ventricular enlargement, left ventricular systolic and diastolic dysfunction, and mild mitral and moderate tricuspid regurgitation.

**Figure 1:**
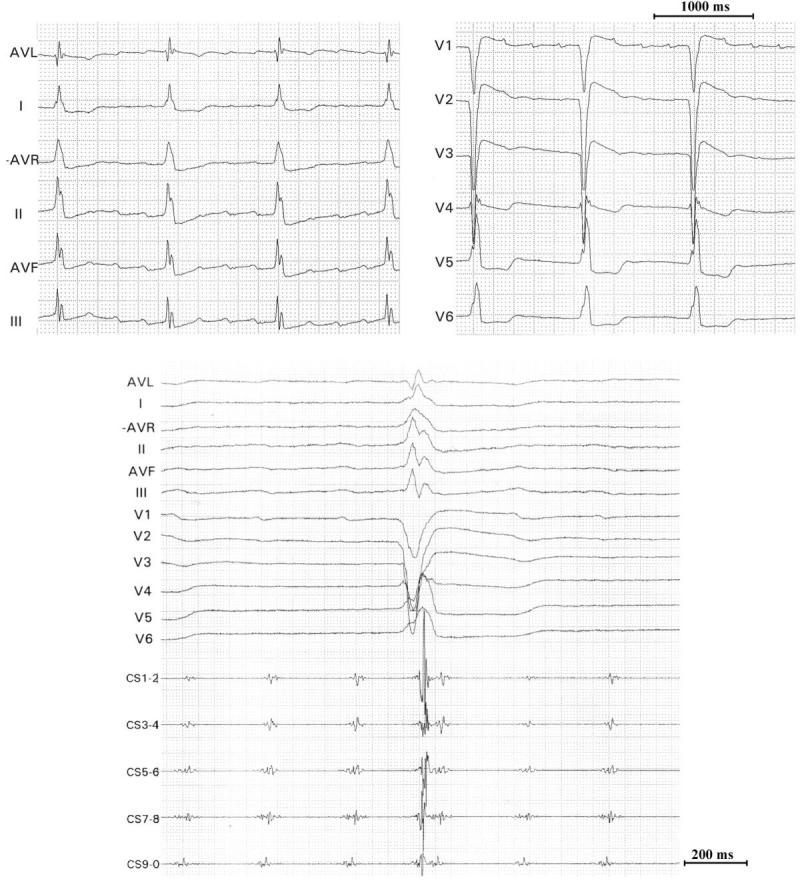
Twelve-lead ECG and intracardiac recordings during tachycardia. Note the positive p waves in lead V1. RF = bipolar electrograms from the ablation catheter; CS = coronary sinus.

At the time of the third EP study, a decapolar catheter was placed in the coronary sinus (CS) and a bipolar catheter was positioned in the right ventricle. An 8-French deflectable quadripolar irrigated-tip catheter (Cordis-Webster, Thermocool, F-curve) was used for mapping and ablation. Mapping was performed using the Carto system (Carto; Biosense Inc). RF current was delivered through a Cordis Stockert generator between the 4-mm tip of the catheter and an adhesive patch on the posterior chest wall. Ablation was performed in temperature-controlled mode with a target temperature of 50°C and a power limit of 50 W. Saline was infused at a rate of 3 ml/min between ablations and 30 ml/min during RF current delivery. After obtaining a right atrial activation map with an electroanatomical reconstruction of the right atrium during proximal CS pacing, a regular tachycardia, with ECG morphology similar to the clinically documented tachycardia, was induced with burst pacing (Figure 1). The CS electrograms were of low amplitude, with electrograms in CS 9-10 starting 23 ms before electrograms in CS 1-2.

**Figure 2:**
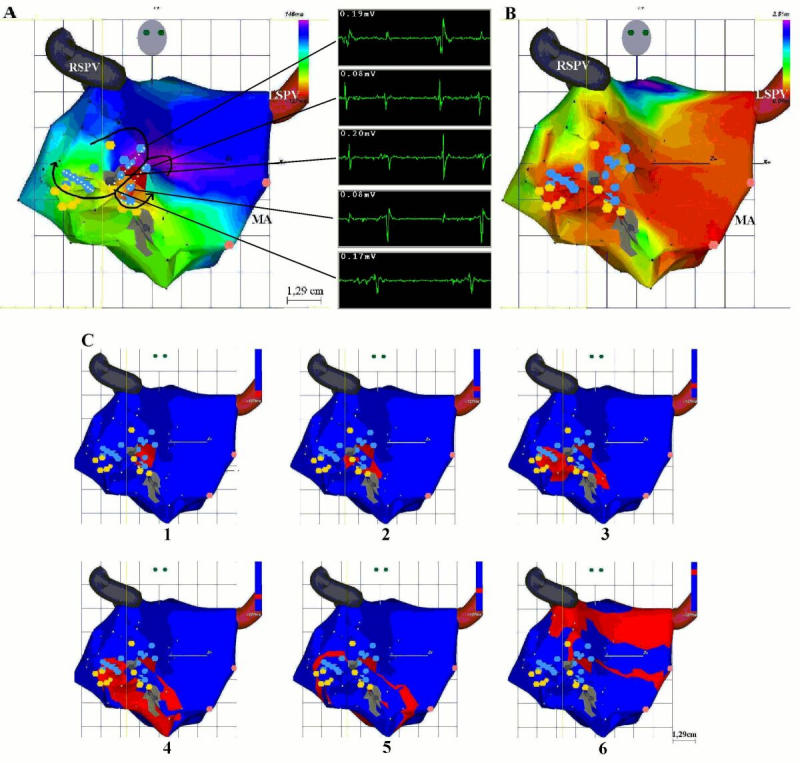
(**A**) Right anterior oblique projection of the activation map of the left atrium during tachycardia. Insets display local electrograms. The entire cycle length of the tachycardia was mapped within a low voltage zone containing double- and fragmented potentials (blue and yellow dots respectively) and electrically silent areas. We hypothesized that the common channel is bordered by two lines of double potentials (parallel white dotted lines) and a discrete scar (voltage 0.04 mV, medial gray area). One loop revolves medially around a third, transverse, line of double potentials (third white dotted line) and then propagates through a channel bordered by this line and the ostium of the right inferior pulmonary vein. The second loop revolves laterally through a channel bordered by the lateral line of double potentials and a second, lower scar (voltage 0.03-0.05 mV, lower gray area). Both groups of points exhibiting fragmented potentials, situated, the first in the lateral channel, the second inferior to the transverse line of block, have close activation times (-50 to -70 mV). The first component of the isolated double potential point situated medially to the upper scar, as well as points situated laterally to the lower scar, exhibit close activation times also; nevertheless they are activated 20-40 ms later than the rest of the fragmented potential points, thus further confirming the bifurcation of the activation front at the exit from the common channel. The left superior pulmonary vein (LSPV) and the right superior pulmonary veins (RSPV) are depicted by red and dark gray tubular icons, respectively. Pink dots represent points situated on the mitral annulus (MA). (**B**) Bipolar voltage map shows that the entire circuit is confined to a larger low voltage area. (**C**) Biosense propagation map depicting activation of the left atrium viewed in a left anterior oblique projection.

Sequential mapping of the right atrium during tachycardia revealed a nonreentrant activation pattern with an early septal area. Mapping of the left atrium was performed through transeptal catheterization. Two discrete scars (areas with bipolar voltage ≤0.05 mV) (1-3) along with adjacent points displaying fragmented or double potentials were located on the left anterior wall. They were confined to a larger region of low voltage (≤0.5 mV). The entire cycle length of the tachycardia was recorded within this region and the activation map was consistent with a figure-8 circuit. We hypothesized that activation propagated craniocaudally, through a common channel bordered by two lines of double potentials in its upper portion (Figure 2). In its inferior part this channel was bordered medially by a discrete scar. One loop then revolved medially, around a transverse line of double potentials and anterior to the ostium of the right inferior pulmonary vein. The second loop revolved laterally, through a channel bordered by the first line of double potentials and the second, lower, scar. A fourth possible channel appeared between the transverse line of double potentials and the medial scar. In order to avoid terminating or transforming the tachycardia or inducing atrial fibrillation, entrainment was not performed in this patient.

Ablation targeted the common isthmus (isolated RF applications) and both the common channel and the medial turnaround of the circuit (point-by-point and dragging technique, aiming also to transect the possible fourth channel). Both attempts failed due to poor catheter stability. With the aim of continuing the ablation procedure under coronary sinus pacing, we attempted to interrupt the tachycardia by overdrive pacing, but this maneuver led to transformation into a second tachycardia with a CL of 190 ms (not shown in this paper). This tachycardia was DC-converted to sinus rhythm and the procedure continued afterwards under CS pacing. Nevertheless, the same technical difficulties further precluded the successful ablation of the circuit. Remapping the ablation points confirmed persistence of electrograms with the same configuration and the procedure was interrupted (long procedure time).

## Discussion

Tachycardias with figure-8 activation pattern have been described both in the right and left atrium, most frequently in patients who have undergone surgery for congenital heart defects and valve disease [1-7]. As with other incisional tachycardias, the electrophysiological substrate is provided by the presence of areas of low voltage and depressed conductivity, electrically silent zones and lines of block that are attributable to an insufficient myocardial protection of cardioplegia, atriotomies and cannulating sites [3,4,8]. In the right atrium the loops are often disposed around the tricuspid annulus and lateral atriotomies, while in the left atrium the geometry seems more variable, with loops rotating around mitral annulus, electrically unexcitable areas, or the ostia of the pulmonary veins [1,2,4,5].

It is conceivable that a figure-8 activation pattern may be encountered in both reentrant and focal tachycardias [9]. The latter scenario supposes the existence of a discharging focus inside a protected isthmus having a zone of slow conduction at one of its extremities. Entrainment is therefore of particular interest in such tachycardias since its demonstration may relatively simply differentiate between a reentrant and a focal mechanism.  Furthermore, a post-pacing interval equal to tachycardia cycle length indicates a site within the reentry circuit while supplementary demonstration of a short stimulus-to-P wave interval points out the exit of the protected isthmus. However, one of the main shortcomings of entrainment mapping is the possibility of interrupting or transforming the tachycardia during rapid pacing. This aspect is of particular concern in patients with left atrial tachycardias [4]. Therefore, some authors prefer to delineate the circuit by performing a high-density activation mapping of the entire cavity avoiding thus entrainment [1-3]. In this case, the reentrant nature of the tachycardia as well as the configuration of the circuit are finally proven by successfully interrupting the loops during ablation (Mines’ test). RF current may be delivered either in the common isthmus or separately for each of the two loops (in the latter case, sometimes with an instantaneous ECG transformation of the tachycardia after the interruption of the first loop) [1].

We have found in the literature 5 patients with atrial tachycardias and a remote history of isolated aortic valve replacement  [2,5-7]. All had macroreentrant tachycardias (4 in the left atrium, 1 in the right atrium) and 1 presented a concomitant focal tachycardia. Among patients with left tachycardias, data regarding activation map and/or ablation were available only in two cases. In the first case, described by Jais et al. [2], although no complete electro-anatomical map was obtained, the tachycardia was successfully ablated by drawing a line between the ostium of the right superior pulmonary vein and the mitral annulus.  In the second case, reported by Ouyang et al. [5], mapping revealed a figure-8 activation pattern with one loop situated in the anterior left atrial wall and the other one in the roof of the same atrium. Curative ablation was performed at the shared channel identified by activation and entrainment mapping.

In this report we describe a figure-8 tachycardia originating in the left anterior atrial wall in another patient with previous aortic valve replacement. The full cycle length of the tachycardia was mapped inside a low voltage zone containing electrically silent areas and multiple sites exhibiting  double- or fragmented potentials. This fact suggests a severely affected anatomical substrate. In order to avoid terminating or transforming the tachycardia, as reported during the previous two EP studies in this patient, we did not perform entrainment. Ablation targeted what was regarded as the common and medial channels (including the possible channel between the medial scar and the transverse line of double potentials) and, presumably, in order to avoid other secondary circuits, it should have been continued in the lateral channel as well. Tachycardia interruption along with its noninducibility after severance of these channels would have supported our hypothesis concerning the electrophysiological mechanism and the configuration of the circuit. However the instability and difficult maneuverability of the catheter, probably explained by the anatomical position of the targeted area, precluded a transmural ablation. Additional factors contributing to this outcome might be the structural changes of the anatomical substrate and the fact that we targeted first the common and medial channels. Shah et al. [1]. noted that in the case of right figure-8 tachycardias, catheter stability in the region of the shared isthmus may be problematic.

This report confirms that patients with previous aortic valve replacement may have areas with abnormal electrophysiology in the left atrium that favor complex atrial tachycardias.  Participation of the left anterior atrial wall in such tachycardias (as showed in the patient presented by Ouyang et al. and in this report) may be related to mechanical trauma during surgery (due to the proximity of this region to aortic root) as well as to intra- and postoperative atrial ischemia. In cases of figure-8 activation patterns, confirmation of the tachycardia mechanism and geometry of the circuit requires detailed activation mapping combined with entrainment and/or a successful ablation strategy (defined as modification/interruption of the tachycardia due to a transmural lesion with impossibility of reinitiating the same tachycardia).
